# Integrated analysis of gene expression and DNA methylation datasets identified key genes and a 6-gene prognostic signature for primary lung adenocarcinoma

**DOI:** 10.1590/1678-4685-GMB-2020-0465

**Published:** 2021-11-15

**Authors:** Jing Meng, Lei Cao, Huifang Song, Lichun Chen, Zhiguo Qu

**Affiliations:** 1 Inner Mongolia People’s Hospital, Department of Stomatology, Hohhot, China.; 2 Inner Mongolia People’s Hospital, Department of Clinical Medical Research Center, Hohhot, China.; 3 Inner Mongolia People’s Hospital, Department of Respiratory and Critical Care Medicine, Hohhot, China.

**Keywords:** Lung adenocarcinoma, prognosis, signature, overall survival, risk score

## Abstract

Lung adenocarcinoma (LUAD) is the main subtype of non-small cell lung cancer with a poor survival prognosis. In our study, gene expression, DNA methylation, and clinicopathological data of primary LUAD were utilized to identify potential prognostic markers for LUAD, which were recruited from The Cancer Genome Atlas (TCGA) database. Univariate regression analysis showed that there were 21 methylation-associated DEGs related to overall survival (OS), including 9 down- and 12 up-regulated genes. The 12 up-regulated genes with hypomethylation may be risky genes, whereas the other 9 down-regulated genes with hypermethylation might be protective genes. By using the Step-wise multivariate Cox analysis, a methylation-associated 6-gene (consisting of CCL20, F2, GNPNAT1, NT5E, B3GALT2, and VSIG2) prognostic signature was constructed and the risk score based on this gene signature classified patients into high- or low-risk groups. Patients of the high-risk group had shorter OS than those of the low-risk group in both the training and validation cohort. Multivariate Cox analysis and the stratified analysis revealed that the risk score was an independent prognostic factor for LUAD patients. The methylation-associated gene signature may serve as a prognostic factor for LUAD patients and the represent hypermethylated or hypomethylated genes might be potential targets for LUAD therapy.

## Introduction

Lung adenocarcinoma (LUAD) is one major subtype of non-small-cell lung cancer (NSCLC) with high mortality (Siegel et al., 2015; Gharibvand et al., 2017). Because of being asymptomatic in the early phase and the delay of diagnosis, the 5-year survival rate is 10.3% in the patients with LUAD (Li et al., 2016). In view of this, it is urgent to develop a reliable biomarker to predict the prognosis of LUAD.

DNA methylation is an epigenetic process involving the addition of a methyl group to DNA. The methylation of DNA has been demonstrated to play an important role in a variety of cellular processes and disordered methylation patterns have been shown to associate with several human diseases, including cancer. Because of the stability, reversibility, and easy detectability, DNA methylation has obtained clinical attention as a novel biomarker for diagnosis and prognosis of cancer (Hao et al., 2017; Xu *et al*., 2017), including lung cancer (Brock et al., 2008). DNA methylation in cancer always occurs in the CpG islands that were presented in the promoters of a gene (Yamashita et al., 2018). As a result, these methylated CpG sites could affect the activation of the promoter and control the expression of the corresponding gene. Typically, high methylation of a gene always inhibits its expression, but in some cases, high methylation has been observed to promote gene expression. Studies have shown that alterations in the expression of methylation-related genes are common in the development and progression of tumors (Sheikhnejad et al., 2013; Swm et al., 2021). Furthermore, methylation-associated genes could be used to predict the prognosis of several cancer types. Although the methylation-associated genes have been demonstrated to be altered in LUAD tissue (Selamat et al., 2012; Bjaans et al., 2016; Pu et al., 2016), the prognosis value of these genes has not been well studied in LUAD.

In the current study, an integrated analysis of gene expression and DNA methylation datasets from the TCGA database was performed to identify methylation-associated prognostic genes for LUAD. A methylation-associated 6-gene signature was constructed and validated, which might contribute to improving the prognosis of LUAD patients, and might be potential targets for LUAD therapy.

## Material and Methods

**Figure 1 -:**
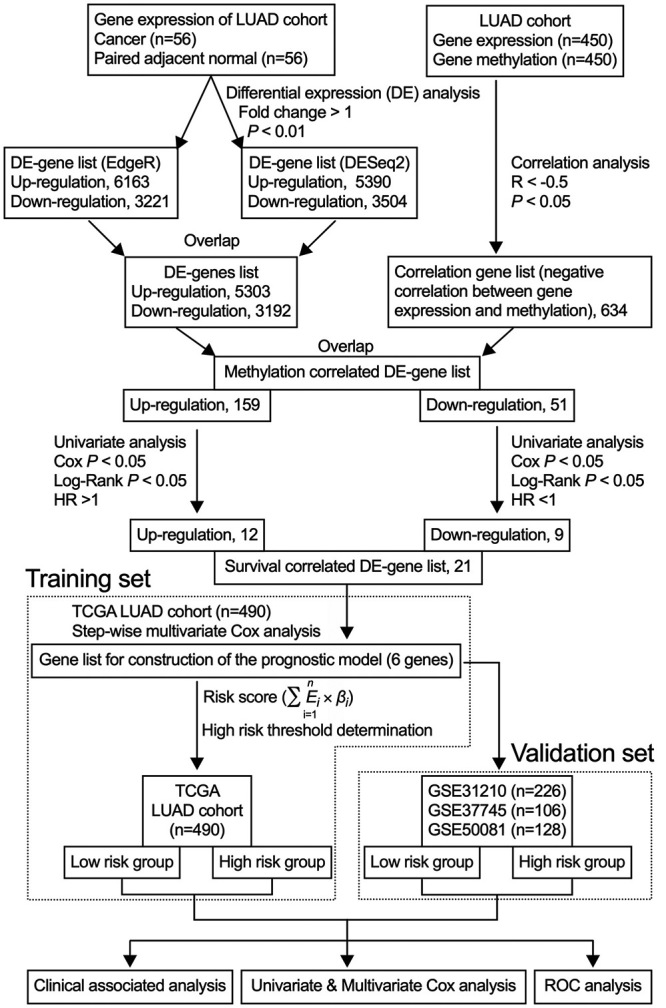
Flow diagram of our study. In parentheses are the numbers of patients in each cohort. All data analyses were conducted in R.

All data analyses were conducted relying on R (http://www.r-project.org/, version 3.5.1). The analysis process is exhibited in Figure 1 as a flow chart.

## Data source

Level 3 gene expression (RNA-seq) data, DNA methylation data, and the corresponding clinical information of the primary LUAD patients were retrieved from the TCGA database (https://portal.gdc.cancer.gov) in September, 2018. The detailed clinical data are shown in Table 2. Methylation data were based on the Illumina Infinium Human Methylation 450k BeadChip. A total of 490 primary LUAD samples were available and selected for further analysis. Among these 490 patients, 56 patients had paired adjacent-normal samples and 450 patients had the DNA methylation data. The expression profiles of LUAD patients under the accession number GSE31210 (Okayama et al., 2012), GSE50081 (Der et al., 2014), and GSE37745 (Botling et al., 2013) from the Gene Expression Omnibus (GEO) database (http://www.ncbi.nlm.nih.gov/gds/?term=) were downloaded as independent validation datasets. There were 226 patients in GSE31210, 106 patients in GSE37745, and 128 patients in GSE50081.

## Screening for differentially expressed genes (DEGs)

EdgeR (Robinson *et al*., 2009) and DESeq2 (Anders and Huber, 2010) Bioconductor packages of R were utilized to evaluate the DEGs between tumor and 56 paired adjacent-normal samples. Then, Benjamini and Hochberg approach was utilized to adjust the P values to false discovery rates (FDR) (Benjamini *et al*., 2001). The DEGs were identified based on |logarithmic fold change| >1, and FDR < 0.01, respectively. Volcano plots and scatter plots were generated using the ggplot2 package (http://ggplot2.org/), Venn Diagrams were plotted using the VennDiagram package (Chen and Boutros, 2011), while heatmaps were created using the pheatmap package (https://cran.r-project.org/web/packages/pheatmap/index.html).

## Correlation analysis between RNA-seq and DNA methylation

A correlation of gene expression and DNA methylation was estimated using Pearson’s correlation methods. The correlation coefficient (R) < - 0.5 and P < 0.05 were used as the threshold for obtaining a list of genes in which gene expression was inversely correlated with methylation.

## Identification of prognosis-related signatures and calculating risk score

The intersections of up-regulated/down-regulated genes and gene list of negative correlation between gene expression and DNA methylation levels were selected as candidates for survival analysis. Then, a univariate Cox model was applied to determine the relationship between the expression level of each candidate DEGs and OS in LUAD patients to investigate which DEGs could be served as prognostic predictors for LUAD. After that, only the DEGs with a P value < 0.05 and hazard ratio (HR) > 1 for up-regulated genes or HR < 1 for down-regulated genes were screened out and fitted into a step-wise multivariate Cox regression to construct the gene signature. HR was utilized to determine the risky genes (HR > 1) and protective genes (HR < 1). Subsequently, the risk score for each patient was computed using the following equation:

$$Risk\;score\;=\;\sum_{i=1}^{n}E_{i}*\beta_{i}$$

where “n” is the number of the selected genes, “Ei” stands for the expression level of gene i, and “βi” represents the coefficient of gene i.

Patients were classified into low- and high-risk groups according to the median risk score (Zhou et al., 2016). Meanwhile, the prognostic performance of the risk score was measured using the time-dependent receiver operating characteristic (ROC) curves by calculating the area under the curve (AUC) using the R package “survivalROC” (Heagerty et al., 2000). The defining point set up by 1-, 2-, 3-, 4- and 5-year time-dependent ROC curve analysis was employed to assess the predictive value of the risk score for time-dependent outcomes (Heagerty *et al*., 2000). Survival curves in the low- and high-risk groups were plotted by means of the Kaplan-Meier methods and the differences in the survival time between the two groups were compared using the Log-Rank test and Cox regression analysis (Jones and Crowley, 1989).

## Cox regression analysis of the prognostic signature and other clinical parameters

Influences of various variables including risk score, age, gender, and stage on OS were evaluated by univariate and multivariate Cox proportional hazard regression models.

## Results

### Identification of methylation associated DEGs

**Figure 2 -:**
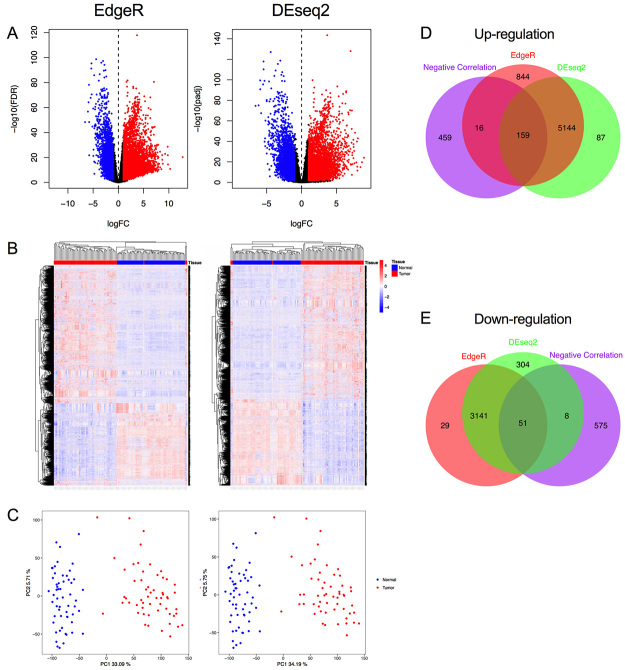
Identification of methylation-associated differentially expressed genes (DEGs) in lung adenocarcinoma (LUAD). (**A**) Volcano plots of DEGs analyzed by edgeR and DESeq2. (**B)** Heatmaps of DEGs identified by edgeR and DESeq2 **(C)** Principal component analysis (PCA) of the DEGs identified by edgeR and DESeq2. (**D)** Overlapping of up-regulated DEGs and the genes list negatively correlated with the DNA methylation. (**E**) Overlapping of down-regulated DEGs and the genes list negatively correlated with the DNA methylation.

To screen out the DEGs between the LUAD and the paired adjacent normal samples, both EdgeR and DEseq2 packages were used. A total of 9384 DEGs were detected by using the EdgeR package according to the threshold of fold change > 1 and an FDR value < 0.01, of which 6163 were up-regulated and 3221 were down-regulated. Meanwhile, 8894 DEGs were identified by using the DESeq2 package, of which 5390 were up-regulated and 3504 were down-regulated. The distribution of the DEGs identified by both EdgeR and DEseq2 were shown using volcano plots (Figure 2A). Unsupervised hierarchical clustering analysis showed that these DEGs could distinguish LUAD samples and the adjacent normal samples (Figure 2B). Similarly, the PCA analysis also suggested that these DEGs could separate samples into LUAD and normal (Figure 2C). Finally, a total of 8495 DEGs were screened out (5303 up- and 3192 down-regulated) by overlapping the DEGs extracted by EdgeR and DEseq2 package.

To identify the methylation associated genes, we analyzed the correlation between the gene expression and the level of DNA methylation. Based on the predefined criteria (R< -0.5 and P < 0.05), a total of 634 methylation associated genes were extracted. Subsequently, the common part between the 634 methylation associated genes and the 8495 DEGs were extracted. Ultimately, 210 methylation-associated DEGs were identified for prognosis analysis, of which 159 was up-regulated (Figure 2D) and 51 was down-regulated (Figure 2E).

### Identification of risky and protective genes

To evaluate the prognostic value of the methylation-associated DEGs, a univariate Cox regression analysis was conducted to investigate the correlation between the expression level of these methylation associated DEGs and the OS of the LUAD patients. Consequently, 21 methylation-associated DEGs (12 up- and 9 down-regulated) were found to be significantly associated with the OS. As shown in Figure S1A and S1B, the HRs of these 12 up-regulated DEGs were more than 1 (risky genes), while those of the 9 down-regulated DEGs were less than 1 (protective genes). All these 21 DEGs showed a negative correlation between the DNA methylation status and the gene expression level (Figure S2). The 12 up-regulated DEGs with hypomethylation might be risky genes (Figure S2A), whereas the other 9 down-regulated DEGs with hypermethylation might be protective genes (Figure S2B).

### Identification of a 6-gene prognostic signature and validation of the risk scoring system based on this gene signature

Subsequently, a step-wise multivariate Cox model was used to conduct a gene signature. Ultimately, a 6-gene signature (including CCL20, F2, GNPNAT1, NT5E, B3GALT2, and VSIG2) was developed (Table 1). Among these 6 genes, 4 genes (CCL20, F2, GNPNAT1, and NT5E) were unfavorable genes (HR > 1) and the 2 genes (B3GALT2, and VSIG2) were favorable genes (HR < 1).

**Table 1 -:** Univariate and multivariate analysis of the 6 genes for constructing the prognostic signature.

Gene symbol	Ensembl ID	Gene type	Chromosomal position	Univariate analysis		Multivariate analysis	
				HR (95% CI)	P	HR (95% CI)	P
CCL20	ENSG00000115009	Protein coding	Chr2: 227805739-227817564	1.11(1.02-1.20)	1.50E-02	1.12(1.03-1.22)	7.00E-03
F2	ENSG00000180210	Protein coding	Chr11: 46719180-46739506	1.13(1.06-1.21)	2.10E-04	1.13(1.05-1.21)	8.20E-04
GNPNAT1	ENSG00000100522	Protein coding	Chr14: 46719180-52791668	1.65(1.34-2.04)	3.50E-06	1.38(1.05-1.80)	1.90E-02
NT5E	ENSG00000135318	Protein coding	Chr6: 46719180-85495791	1.17(1.06-1.31)	3.10E-03	1.23(1.11-1.37)	1.60E-04
B3GALT2	ENSG00000162630	Protein coding	Chr1: 46719180-193186654	0.85(0.77-0.95)	4.10E-03	0.89(0.79-1.00)	4.40E-02
VSIG2	ENSG00000019102	Protein coding	Chr11: 46719180-124752238	0.88(0.82-0.95)	5.20E-04	0.87(0.79-0.97)	1.20E-02

HR: hazard ratio; CI: confidence interval

**Figure 3 -:**
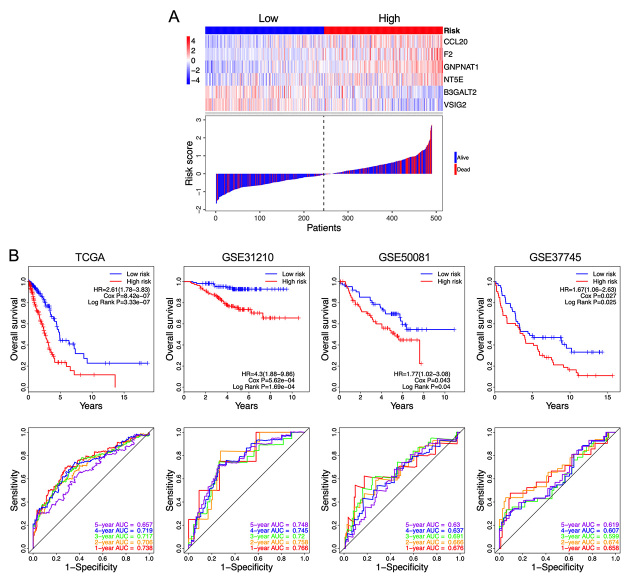
Construction and validation of the methylation-associated gene signature. (**A)** The expression patterns of the 6 genes in the high-risk and low-risk group, and the survival status of each LUAD patient in the TCGA data set. The black dotted line is the median risk score, which is utilized to divide patients into the high-risk group and low-risk group. (**B)** Kaplan-Meier and ROC analysis of the survival prediction performance of the risk score which was based on the methylation-associated gene signature. TCGA was used as a training set, and GSE31210, GSE50081, and GSE37745 were used as validation sets.

For each LUAD patient, the risk score was calculated based on the gene expression level and the Cox regression coefficient. According to the threshold of median risk score, 490 patients were divided into a high-risk group and a low-risk group based on the median risk score. The expression pattern of the 6 genes and the survival situation of LUAD patients in the high-risk group and low-risk group were displayed in Figure 3A. From this figure, we found that the mortality rate in the high-risk group was higher, relative to that in the low-risk group. 

To investigate the prognostic value of the risk score based on the 6-gene signature in the TCGA LUAD dataset, a univariate analysis was performed. The Kaplan-Meier curves suggested that the OS time of patients in the high-risk group was shorter than that of the low-risk group (Figure 3B, cox P = 8.42e-07, log-rank P = 3.33e-07), which implicated that the high-risk score was a poor prognostic factor for patients with LUAD (HR = 2.61, 95% CI= 1.78-3.83). The prognostic capacity of the risk score was investigated by calculating the AUC value of the ROC curves. The time-dependent ROC curves for 1-, 2-, 3-, 4, and 5-year survival prediction are listed in Figure 3B, with an AUC of 0.738, 0.706, 0.717, 0.719, and 0.657, respectively, demonstrating that the risk score had a high specificity and sensitivity in predicting of OS. 

To investigate the reliability of the risk score for prediction of OS, 3 expression profile datasets including GSE31210, GSE50081 and GSE37745 were used for validation. The results implicated that all patients in the high-risk group had a shorter OS than those in the low-risk group (GSE31210: HR = 4.3, 95% CI = 1.88-9.86, Log-Rank P =1.69e-04; GSE50081: HR = 1.77, 95% CI = 1.02-3.08, Log-Rank P = 0.04; GSE37745: HR = 1.67, 95% CI = 1.06-2.63, Log-Rank P = 0.025) (Figure 3B). The time-dependent ROC curves showed that the 1-, 2-, 3-, 4-, and 5-year AUC values were 0.766, 0.758, 0.72, 0.745, and 0.748 in GSE31210, 0.676, 0.666, 0.691, 0.637, and 0.63 in GSE50081, 0.658, 0.674, 0.599, 0.607, and 0.619 in GSE37745, respectively (Figure 3B), demonstrating a reliable performance for predicting OS.

**Figure 4 -:**
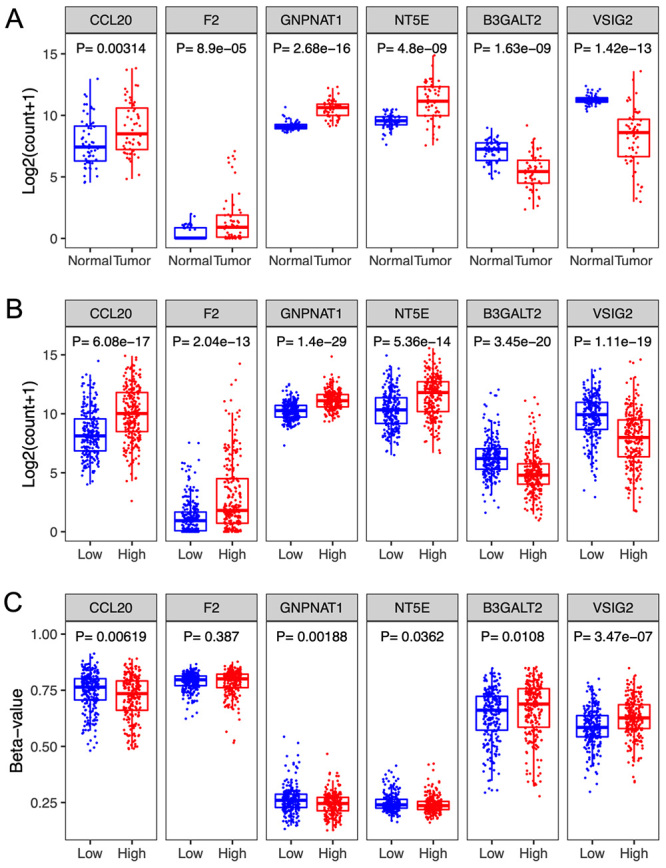
Expression patterns of methylation-associated gene signature. The expression level of the 6 genes in LUAD and paired adjacent normal tissues (**A**), and in high-risk and low-risk groups (**B**). (**C**) DNA methylation levels (Beta-value) of the 6 genes in high-risk and low-risk groups.

The gene expression pattern and the DNA methylation levels of the 6 genes are shown in Figure 4. The expression level of CCL20, F2, GNPNAT1 and NT5E in the tumor samples was significantly higher than that in normal tissues, while the expression level of B3GALT2, and VSIG2 displayed an opposite expression pattern (Figure 4A). Similarly, the expression of CCL20, F2, GNPNAT1 and NT5E was significantly higher in the high-risk group compared to the low-risk groups, but the expression of B3GALT2 and VSIG2 in the high-risk group was lower than that of the low-risk group (Figure 4B). A comparison of the DNA methylation levels of these 6 genes between high- and low-risk groups was performed (Figure 4C). Of note, the methylation level of 3 genes (CCL20, GNPNAT1, and NT5E) was down-regulated in the high-risk group compared with the low-risk group (all P < 0.05). In contrast, the methylation levels of the 2 genes (B3GALT2 and VSIG2) were up-regulated in the high-risk group (both P < 0.05). However, no difference in DNA methylation level of F2 was observed in the two groups (P > 0.05).

### The risk score is an independent survival predictive factor

To investigate the prognostic factors for LUAD patients, a univariable Cox analysis was carried out based on the selected variables including age, gender, stage, and risk score. The results showed that stage II (HR = 2.68, 95% CI = 1.68-4.26, P = 3.20E-05), stage III (HR = 4.39, 95% CI = 2.77-6.98, P = 3.80E-10), stage IV (HR = 3.22, 95% CI = 1.64-6.32, P = 6.70E-04), and high-risk score (HR = 2.61, 95% CI = 1.78-3.83, P = 8.40E-07) were significantly correlated with poor OS of LUAD patients (Table 2). 

To measure whether the risk score was independent of other clinical features, multivariable Cox analysis was implemented. The results showed that age (HR = 1.62, 95% CI = 1.11-2.37, P = 1.20E-02), stage II (HR = 2.60, 95% CI = 1.62-4.18, P = 7.70E-05), stage III (HR = 3.37, 95% CI = 2.08-5.43, P = 7.00E-07), stage IV (HR = 3.69, 95% CI = 1.86-7.32, P = 2.00E-04), and high risk score (HR = 2.30, 95% CI = 1.53-3.46, P = 6.30E-07) were independent prognostic factors for LUAD patients (Table 2). 

**Figure 5 -:**
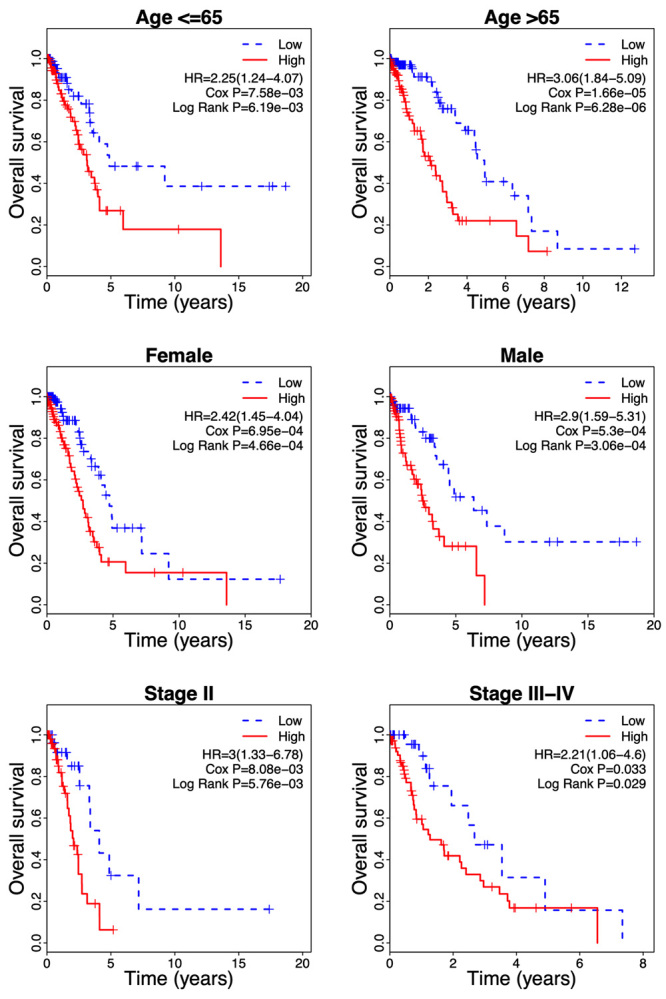
Stratification analysis of the survival prediction performance of the risk score in different clinical parameters of LUAD patients. HR, hazard ratio.

A stratification analysis was further performed based on clinical parameters. Patients in each subgroup including age (<= 65, and > 65), gender (male and female), and stage (II, and III-IV) were separated into the low-risk group and high-risk group according to the median risk score. For all stratified clinical variables, patients in the high-risk group had a shorter survival time, relative to those of the low-risk group (Figure 5, Log-Rank P < 0.05, Cox P < 0.05). Taken together, these findings suggested that the risk score based on the 6-gene signature was an independent survival predictive factor.

**Table 2 -:** Univariate and multivariate Cox regression analysis of the 6-gene signature and overall survival of LUAD patients.

Variables		Patients (N)	Univariate analysis		Multivariate analysis	
			HR (95% CI)	P	HR (95% CI)	P
Age	<=65/>65	237/253	1.35(0.94-1.95)	1.00E-01	1.62(1.11-2.37)	1.20E-02
Gender	Male/Female	265/225	0.93(0.65-1.33)	6.90E-01	0.79(0.55-1.15)	2.20E-01
Stage	I/II	262/115	2.68(1.68-4.26)	3.20E-05	2.60(1.62-4.18)	7.70E-05
Stage	I/III	262/80	4.39(2.77-6.98)	3.80E-10	3.37(2.08-5.43)	7.00E-07
Stage	I/IV	262/26	3.22(1.64-6.32)	6.70E-04	3.69(1.86-7.32)	2.00E-04
Risk score	Low/High	245/245	2.61(1.78-3.83)	8.40E-07	2.30(1.53-3.46)	6.30E-05

HR: hazard ratio; CI: confidence interval

## Discussion

DNA methylation can regulate the gene expression and usually induces tumor suppressor gene silencing and oncogene activation through hyper/hypomethylation (Belinsky, 2004; Vaissiã Re et al., 2008). In this study, we demonstrated for the first time that integrated analysis of gene expression profiles and DNA methylation data could establish methylation-associated gene signature that can be used to predict the survival of LUAD patients. The risk score based on the methylation-associated gene signature exhibited good predictive performance in both TCGA and GEO datasets.

Our methylation-associated gene signature showed comparable sensitivity and specificity to the previous study for survival prediction (Figure S3). In a previous study (Lau et al., 2007), a 3-gene signature classified the patients into two groups and showed different survival times, however, the AUC values of the gene signature were not assessed. Although an 8-gene signature (He and Zuo, 2019) performed better in predicting survival in NSCLC patients, it did not perform as robustly as the gene signature in our study for predicting LUAD. A similar predictive performance was observed between a 7-gene signature (Krzystanek et al., 2016) and our methylation-associated gene signature, nevertheless, the AUC in their study was not calculated. In a 4-gene signature (Cui et al., 2019), the predictive performance based on the TCGA dataset was inferior to our signature, and the AUC values were not validated. Although all signatures were capable of predicting OS, our methylation-associated gene signature was much more robust.

In this methylation-associated gene signature, the expression level of CCL20, F2, GNPNAT1, and NT5E was significantly up-regulated in the LUAD tissue and in the high-risk group. Significantly, the HRs of these 4 up-regulated genes were more than 1, and those methylation levels were hypomethylated, which indicated that these hypomethylated-up-regulation genes are risky genes. Chemokines are responsible for the establishment of the tumor microenvironment, and the infiltration and migration of inflammatory cells and cancer cells (King, 2015). CCL20, a member of CC chemokines, has been observed to mediate the migration of inflammatory cells, thereby involving in metastasis of cancer, including colorectal, pancreatic, or lung cancer (Beider et al., 2009; Brand et al., 2010; Wang et al., 2016). Moreover, Wang *et al*. (2015) have demonstrated that CCL20 is up-regulated in lung cancer, and increased CCL20 is related to poor prognosis. The full name for F2 is coagulation factor II which has been reported to be a prerequisite for lung-cancer-cell-induced platelet aggregation (Heinmöller et al., 1996). Significantly, in some instances, platelet aggregation directly links with the metastatic potential (Tang and Honn, 1995). GNPNAT1 was only reported in prostate cancer, which was suggested to be over-expressed in prostate cancer tissue (Ren et al., 2016) and to be connected with the development of castrationresistant prostate cancer (Kaushik et al., 2016). Growing evidence shows that NT5E is a key regulatory molecule in the development of cancer and is highly expressed in a number of cancers, including NSCLC (Zhu et al., 2017), and silence of NT5E suppresses the cell growth and migration of NSCLC cells (Zhu *et al*., 2017). Significantly, high NT5E expression was an independent predictor of poor prognosis for OS and recurrence-free survival in NSCLC (Inoue et al., 2017). Another two genes identified in our prognostic signature are B3GALT2 and VSIG2 which were down-regulated in the LUAD samples and in the high-risk group in our study. Moreover, those HR values were less than 1, and these two genes were hypermethylated, suggesting genes B3GALT2 and VSIG2 with hypermethylated-down-regulation were protective genes. In a former study, 1 down-regulated gene B3GALT2 was identified among 139 LUAD-specific hypermethylated genes (Yin et al., 2014), which is in line with our results. B3GALT2 is applied to form a prognostic biomarker of carcinoma-associated fibroblasts in NSCLC (Navab et al., 2011). Additionally, carcinoma-associated fibroblasts play a crucial role in maintaining an optimal cancer microenvironment for cell proliferation and survival (Cirri and Chiarugi, 2012; Marsh et al., 2013). VSIG2 has been found to be differentially expressed in endometrial cancer (Shi et al., 2018), and to be significantly associated with bladder cancer risk (Moore et al., 2010). Since the 6-gene signature is established based on the hypomethylation-related risky genes and hypermethylation-related protective genes, it can provide new ideas for methylation-based treatment for LUAD. For example, the methylation strategy of a hypomethylated risky gene, or the demethylation of a hypermethylated protective gene in the signature.

Nevertheless, several disadvantages should be acknowledged in the current study. Firstly, this study is a retrospective study based on previously published datasets, hence, prospective studies should be carried out in the future to remedy the limitations of the retrospective study. Secondly, the functions of these methylation-associated DEGs should be verified based on experimental investigations. Thirdly, clinical studies are needed to further verify the accuracy and application potential of this novel prognostic signature for LUAD patients. 

## Conclusion

The risk score based on the methylation-associated gene signature is an independent survival predictive factor for LUAD patients. The potential clinically applicable methylation-associated gene signature may contribute to improving the prognosis of LUAD patients and the represent hypermethylated or hypomethylated genes might be potential targets for LUAD therapy.

## Supplementary material

The following online material is available for this article:

Figure S1 - Kaplan-Meier survival curves of DEGs.

Figure S2 - Correlation between gene expression and methylation was determined using Pearson’s correlation coefficient (R).

Figure S3 - Kaplan-Meier survival curves and ROC curves of previously published gene signatures.
