# Supplementary material for: Integrated analysis of gene expression and DNA methylation datasets identified key genes and a 6-gene prognostic signature for primary lung adenocarcinoma
Source: Genet Mol Biol. 2021 Nov 15;44(4):e20200465. doi: 10.1590/1678-4685-GMB-2020-0465 (PMC8596225; doi:10.1590/1678-4685-GMB-2020-0465)
Supplement: Figure S1 - [file 1415-4757-GMB-44-4-e20200465-s1.pdf]

Genetics and Molecular Biology
https://doi.org/10.1590/1678-4685-GMB-2020-0465

Supplementary Material to "Integrated analysis of gene expression and DNA methylation datasets identified key genes and a 6-gene prognostic signature for primary lung adenocarcinoma"

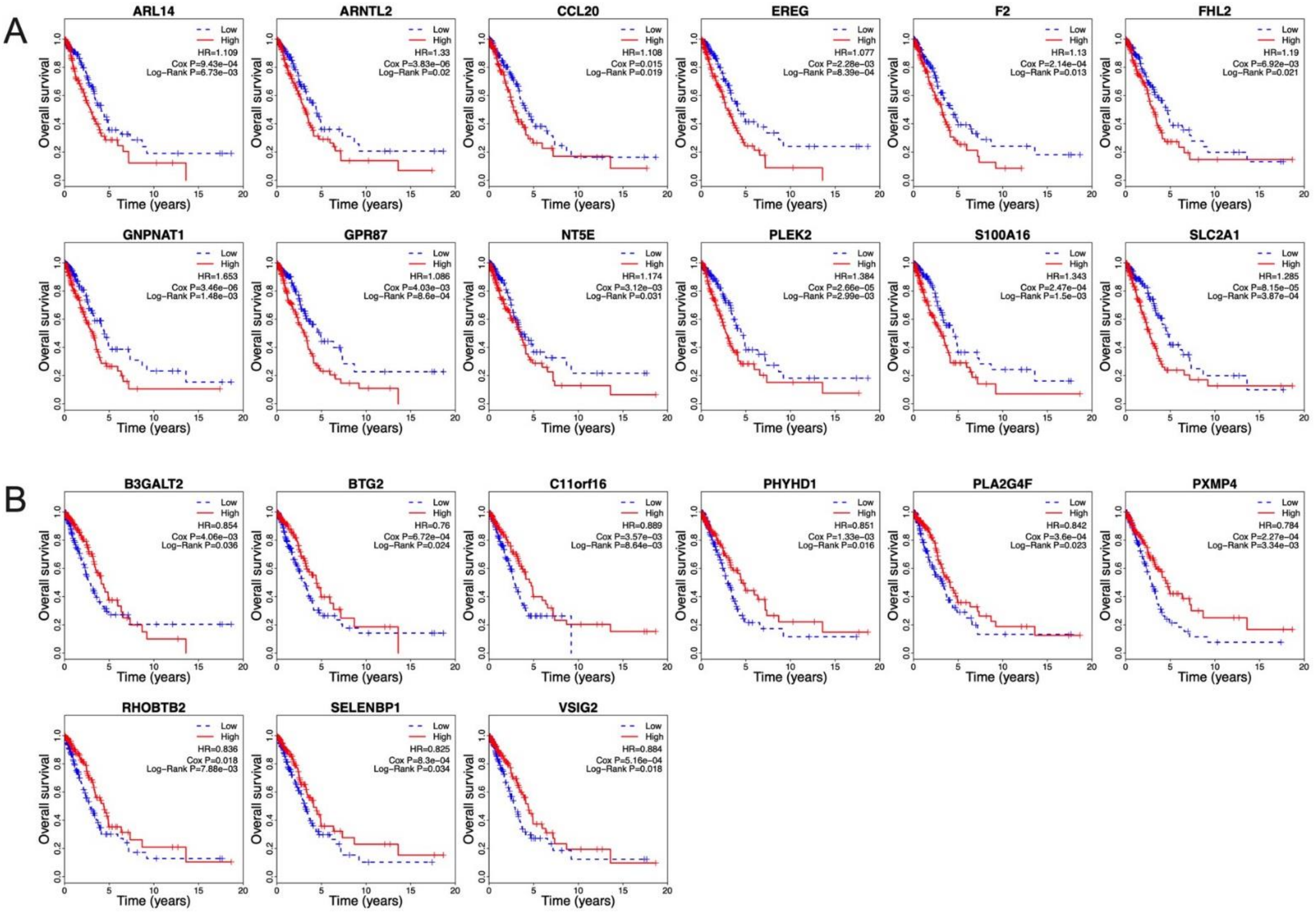

Figure S1 - Kaplan-Meier survival curves of DEGs. The differences in the survival time between the two groups were compared using the Log-Rank test and the Cox regression analysis. (A) Survival curves of 12 up-regulated DEGs. The HR values of these genes were more than 1, which were considered risky genes. (B) Survival curves of 9 down-regulated DEGs. The HR values of these genes were less than 1, which were regarded as protective genes.
