# Supplementary material for: Integrated analysis of gene expression and DNA methylation datasets identified key genes and a 6-gene prognostic signature for primary lung adenocarcinoma
Source: Genet Mol Biol. 2021 Nov 15;44(4):e20200465. doi: 10.1590/1678-4685-GMB-2020-0465 (PMC8596225; doi:10.1590/1678-4685-GMB-2020-0465)
Supplement: Figure S2 - [file 1415-4757-GMB-44-4-e20200465-s2.pdf]

Genetics and Molecular Biology
https://doi.org/10.1590/1678-4685-GMB-2020-0465

Supplementary Material to "Integrated analysis of gene expression and DNA methylation datasets identified key genes and a 6-gene prognostic signature for primary lung adenocarcinoma"

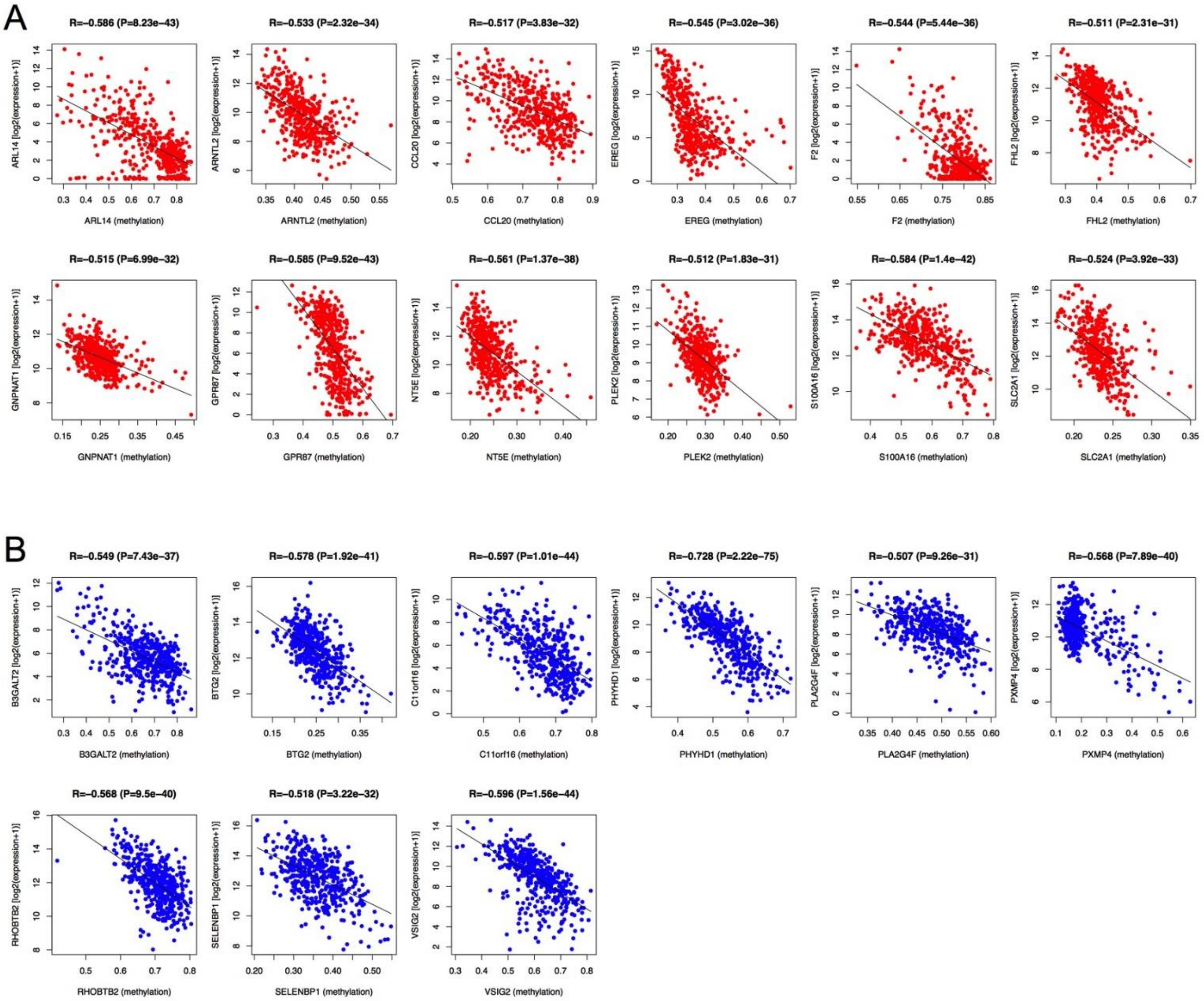

Figure S2 - Correlation between gene expression and methylation was determined using Pearson's correlation coefficient (R). R < -0.5 and P < 0.05 were used as the threshold. (A) Correlation between 12 up-regulated DEGs and DNA methylation. (B) Correlation between 9 down-regulated DEGs and DNA methylation.
