# Supplementary material for: Integrated analysis of gene expression and DNA methylation datasets identified key genes and a 6-gene prognostic signature for primary lung adenocarcinoma
Source: Genet Mol Biol. 2021 Nov 15;44(4):e20200465. doi: 10.1590/1678-4685-GMB-2020-0465 (PMC8596225; doi:10.1590/1678-4685-GMB-2020-0465)
Supplement: Figure S3 - [file 1415-4757-GMB-44-4-e20200465-s3.pdf]

Genetics and Molecular Biology
https://doi.org/10.1590/1678-4685-GMB-2020-0465

Supplementary Material to "Integrated analysis of gene expression and DNA methylation datasets identified key genes and a 6-gene prognostic signature for primary lung adenocarcinoma"

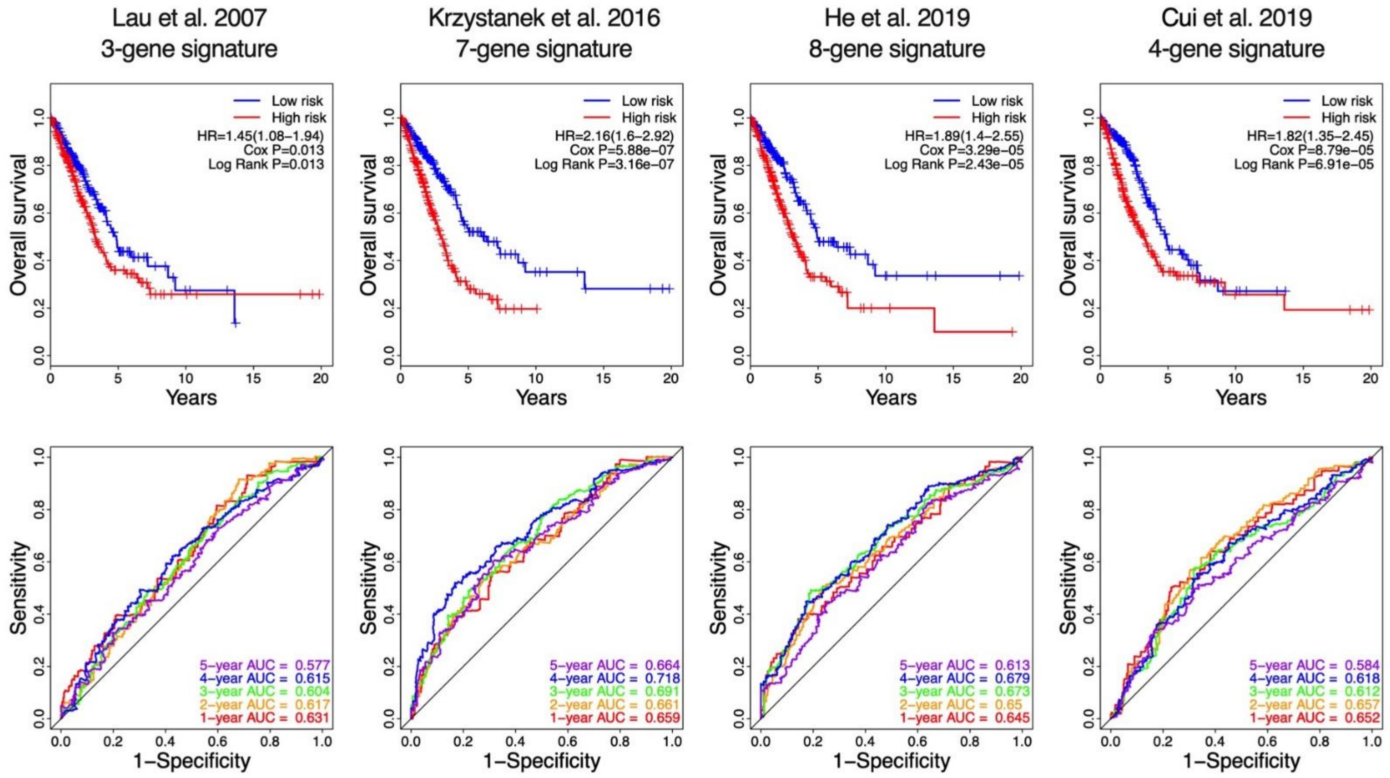

Figure S3 - Kaplan-Meier survival curves and ROC curves of previously published gene signatures.
